# Tumor-Derived Tissue Factor Aberrantly Activates Complement and Facilitates Lung Tumor Progression via Recruitment of Myeloid-Derived Suppressor Cells

**DOI:** 10.3390/ijms18010022

**Published:** 2017-01-19

**Authors:** Xiao Han, Haoran Zha, Fei Yang, Bo Guo, Bo Zhu

**Affiliations:** 1 Institute of Cancer, Xinqiao Hospital, Third Military Medical University, Chongqing 400037, China; hanx310@tmmu.edu.cn (X.H.); zhahaoran@tmmu.edu.cn (H.Z.); 2 Department of Pathogenic Biology, Third Military Medical University, Chongqing 400037, China; yangf129@tmmu.edu.cn; 3 Chongqing Key Laboratory of Tumor Immunotherapy, Xinqiao Hospital, Third Military Medical University, Chongqing 400038, China

**Keywords:** tumor-derived tissue factor, complement, myeloid-derived suppressor cells

## Abstract

The initiator of extrinsic coagulation, tissue factor (TF), and its non-coagulant isoform alternatively spliced TF (asTF) are closely associated with tumor development. In the tumor microenvironment, the role of TF-induced coagulation in tumor progression remains to be fully elucidated. Using TF-knockdown lung tumor cells, we showed that TF is the dominant component of procoagulant activity but is dispensable in the cellular biology of tumor cells. In a xenograft model, using immunohistochemical analysis and flow cytometry analysis of the tumor microenvironment, we demonstrated that TF-induced fibrin deposition, which is correlated with complement activation and myeloid-derived suppressor cell (MDSC) recruitment, is positively associated with tumor progression. C5aR antagonism blunted the effect of TF on tumor progression and decreased MDSC recruitment. In conclusion, our data suggested that in tumor microenvironment, TF-induced coagulation activated the complement system and subsequently recruited myeloid-derived suppressor cells to promote tumor growth, which brings new insights into the coagulation-induced complement activation within the tumor microenvironment during tumor progression.

## 1. Introduction

Cancer-associated hypercoagulation is a major concern in cancer treatment, as subsequent cancer-associated venous thromboembolism events (VTE) increase the mortality of cancer patients [1]. The immunostaining of many cancer tissues from different histological origins revealed high tissue factor (TF) expression in tumor tissues, which has been reported to contribute to the hypercoagulant state of cancer patients [2,3,4]. Physiologically, TF is widely expressed underneath the subendothelial tissue, forming an envelope to prevent any disruption of the endothelium. Once exposed to blood, TF initiates a coagulation cascade through an interaction with Factor VII (FVII) and subsequently cleaves Factor X (FX) to its active form, FXa, which converts prothrombin to thrombin, leading to fibrin deposition as well as clot formation. The overexpression of TF and its non-procoagulant isoform, alternatively spliced TF (asTF), were found both to facilitate tumor progression experimentally and to be associated with poor prognosis clinically, thus leading to worldwide studies of TF and its isoform in cancer therapy [5,6,7,8]. Previous studies mainly focused on the intracellular signaling induced by the TF/FVIIa complex [9,10], the proangiogenesis effect of asTF [5], and clot formation induced by tumor-derived TF in the circulation that was directly linked to cancer-associated VTE [4], but few reported the coagulant states within tumor microenvironment. Recently, researchers demonstrated that in the tumor microenvironment, tumor-derived TF was the major cause of local thrombin generation [11]. Another study from an experimental metastatic model also suggested coagulation induced by tumor-derived TF facilitated premetastatic niche formation in a monocyte/macrophage dependent manner [12]. Nevertheless, in the primary tumor site, the understanding of coagulation induced by TF within tumor microenvironment remains incomplete.

Complement is a genetically conserved system consisting of more than 50 circulating and membrane-bound proteins; it functions as the first barrier in the host immune system, becoming activated upon the recognition of molecular patterns from microorganisms and abnormal host cells [13]. Activation of the complement cascade elicits pro-inflammatory responses, resulting in the regulation of immune cells from both the innate and adaptive branches [14]. In the traditional view, the complement system should be able to fight against tumors, as the terminal complement complex (TCC), also known as the membrane attack complex (MAC) and complement 5b-9 complex (C5b-9), can efficiently induce membrane disintegration in abnormal host cells, including tumor cells [15]. However, recent studies have revealed novel evidence that anaphylatoxin C5a, the proteolytic product of complement C5, facilitated tumor progression by promoting cell proliferation and angiogenesis and shifting the tumor microenvironment to an immune suppressive condition via myeloid-derived suppressor cell (MDSC) recruitment [16,17]. These findings indicate that complement has dual roles in the tumor microenvironment, leading to the possibility of targeting complement components in cancer treatment.

For decades, the complement system was believed to be activated through the Classical pathway, the Alternative pathway, and the Lectin pathway, in which C3 functions as pivot. In 2006, Peter et al. discovered a novel complement cleavage mechanism in an acute lung inflammatory injury model by which thrombin can efficiently cleave C5 in the absence of C3 [18]. This finding was soon extended to a multi-intercommunication between the complement system and the coagulation cascade through in vitro experiments [19]. Later, a study from Conway et al. revealed the details of thrombin-induced complement C5 cleavage, indicating that this catalysis is divided into two steps, which is slower compared to the catalysis of C5-convertase [20]. The potential role of this intercommunication within the tumor microenvironment has yet to be determined. As it is known that cancer is regarded as a chronic disease that produces an inflammatory pattern different from that of an acute disease model, and drawing together the independent evidence that (i) TF is the dominant coagulation factor within the tumor microenvironment [11]; (ii) complement components could be cleaved by coagulant factors [18,19,20]; and (iii) complement facilitates tumor progression [16], an attractive hypothesis arose that coagulation induced by tumor-derived TF within the tumor microenvironment may facilitate tumor progression via the activation of complement.

To prove our hypothesis, we down-regulated TF expression in a lung tumor cell line and evaluated the procoagulant activity of tumor-derived TF in vitro. The effect of TF in tumor cell biology in vitro as well as tumor progression in vivo was also examined. We also examined the coagulation and complement activation state in tumor tissues. We report that down-regulation of TF expression resulted in decreased fibrin deposition as well as complement activation within the tumor microenvironment. We also found decreased MDSC infiltration in tumors with low TF expression, which was dependent on C5aR. This study suggests that tumor-derived TF induces coagulation within the tumor microenvironment, which contributes to complement activation and subsequent MDSC recruitment, leading to tumor progression.

## 2. Results

### 2.1. Tissue Factor Expression Knockdown Reduces the Procoagulant Activity of Lung Tumor Cells

**Figure 1:**
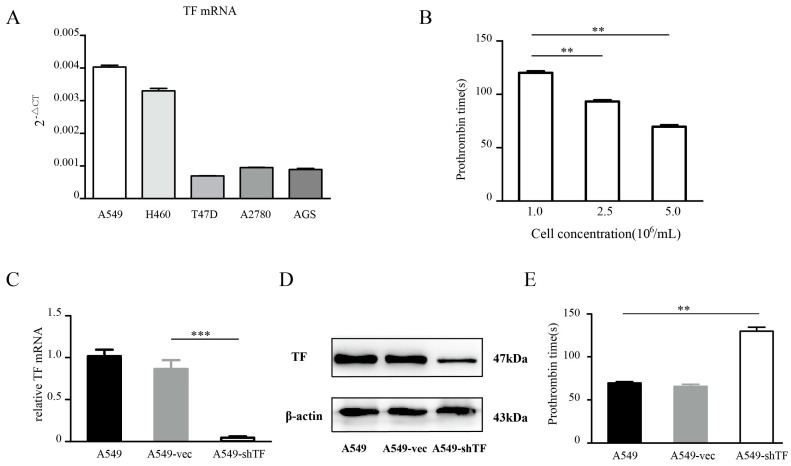
Quantification of tissue factor (TF) and the procoagulant activity of tumor cell. (**A**) TF mRNA was measured in human lung adenocarcinoma cell line A549, breast cancer cell line T47D, ovarian cancer cell line SKOV3, and gastric adenocarcinoma cell line AGS using real-time Polymerase chain reaction (rt-PCR) (*n* = 3); (**B**) A549 cells in a series of cell concentrations were tested for their procoagulant activity by measuring prothrombin time after mixing with recalcificated plasma (*n* = 3); A549 cells were infected by lentiviruses carrying the *shTF* gene and empty control. After cell sorting by flow cytometry, these cells were harvested and confirmed by Polymerase chain reaction (**C**) and Western blot (**D**) (*n* = 3); (**E**) A549 cells, A549-vec cells, and A549-shTF cells were tested for their procoagulant activity using the same protocol as described above (*n* = 3). Data are expressed as mean ± SEM. A549-vec, A549 cells transfected with an empty vector; A549-shTF, A549 cells transfected with shRNA targeting *TF* gene. ** *p* < 0.01, *** *p* < 0.001.

Several tumor cell lines from different tumor types were tested for their TF expression (Figure 1A), and A549 cells were chosen for further procoagulant activity evaluations due to their relatively high TF expression. The procoagulant activity of cells was represented by the prothrombin time (PT), which was measured by mixing A549 cells with citrated human plasma and allowing recalcification to occur. We found that after recalcification, A549 cells could trigger the coagulation cascade in the plasma-cell mixture, and this process was dramatically prolonged when the cell concentration decreased (Figure 1B). Consistent with previous studies in which TF’s procoagulant activity on A549 cells was blocked with an antibody [5,21], we used a short hairpin RNA (shRNA) to genetically interfere with the TF expression of A549 cells to validate the connection between TF and tumor cell procoagulant activity. The efficiency of TF knockdown among A549 cells, A549 cells transfected with an empty vector (A549-vec cells for short), and A549 cells transfected with shRNA targeting *TF* gene (A549-shTF cells for short) was validated at both the mRNA and protein levels (Figure 1C,D). Next, these cells were tested for their procoagulant activity in a series of cell concentrations. A549-shTF cells failed to induce measurable clotting when the cell concentration was below 5 × 10^6^/mL. At a cell concentration of 5 × 10^6^ cell/mL, all three cell lines could successfully induce the plasma-cell mixture to clot, and the PT induced by A549-shTF cells was prolonged by about 2-fold compared with that of A549 cells and A549-vec cells (Figure 1E).

### 2.2. TF Knockdown Does Not Affect the Proliferation and Apoptosis of Lung Tumor Cells

**Figure 2:**
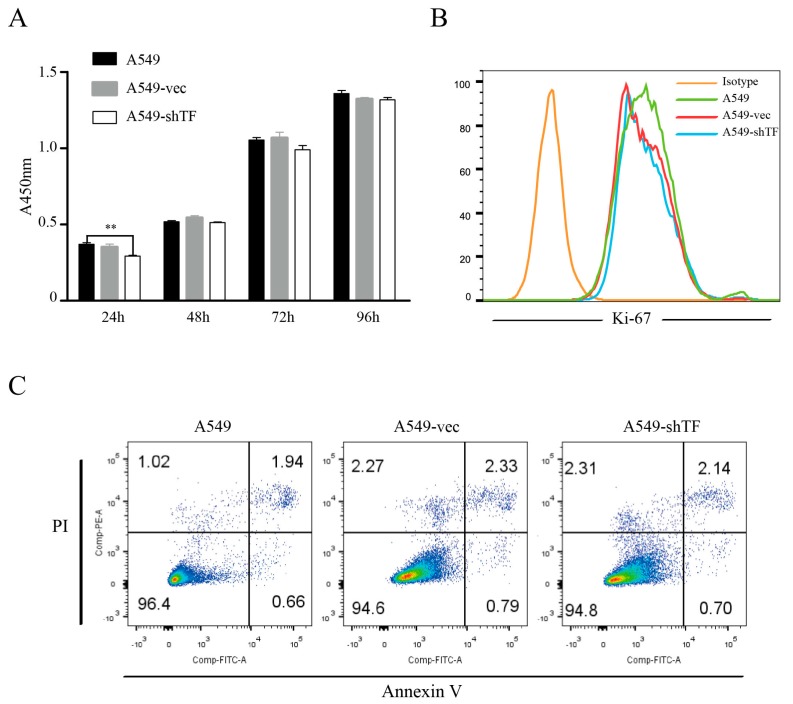
Evaluation of tumor cells proliferation and apoptosis after TF knockdown. (**A**) The proliferation of A549 cells, A549-vec cells, and A549-shTF cells was determined by cell counting kit-8 (CCK-8) assay (*n* = 3); (**B**) the percentage of Ki-67 positive cell in A549 cells, A549-vec cells, and A549-shTF cells were measured by flow cytometry (*n* = 3); (**C**) A549 cells, A549-vec cells, and A549-shTF cells apoptosis were detected by flow cytometry with Annexin V-FITC/PI staining (*n* = 3). PI, Propidium Iodide, ** *p* < 0.01.

The function of TF in cell biology remains controversial. The results from embryo development studies showed that teratomas from TF^−/−^ embryonic stem (ES) cells exhibited equal tumor growth and frequency compared to normal ES cells [22], while another study suggested that blocking TF with an antibody in a xenotransplant tumor model resulted in delayed growth [23]. To assess whether TF knockdown affects the cellular biology of A549 cells, we tested the proliferation ability of A549 cells, A549-vec cells, and A549-shTF cells in vitro. A CCK-8 assay was used to detect cell numbers each day after seeding, and the result showed that although a slightly reduced cell number of A549-shTF cells exists at 24 h compared to A549 cells, these three cells exhibited comparable proliferative ability at the rest time point (Figure 2A). To validate our result, we also measured the Ki-67 level, which reflected the proliferative potential of the cells, and no difference in the proportion of Ki-67^+^ cells was observed among A549 cells, A549-vec cells, and A549-shTF cells (Figure 2B). In addition to the proliferative ability, we also evaluated the apoptosis rate of A549 cells after TF knockdown. Flow cytometric analysis of Propidium Iodide (PI) and annexin V stained-cells showed that the apoptosis rate of A549-vec cells and A549-shTF cells remained equal regardless of TF expression. (Figure 2C).

### 2.3. TF-Facilitated Tumor Growth Is Associated with Local Coagulation Activation

**Figure 3:**
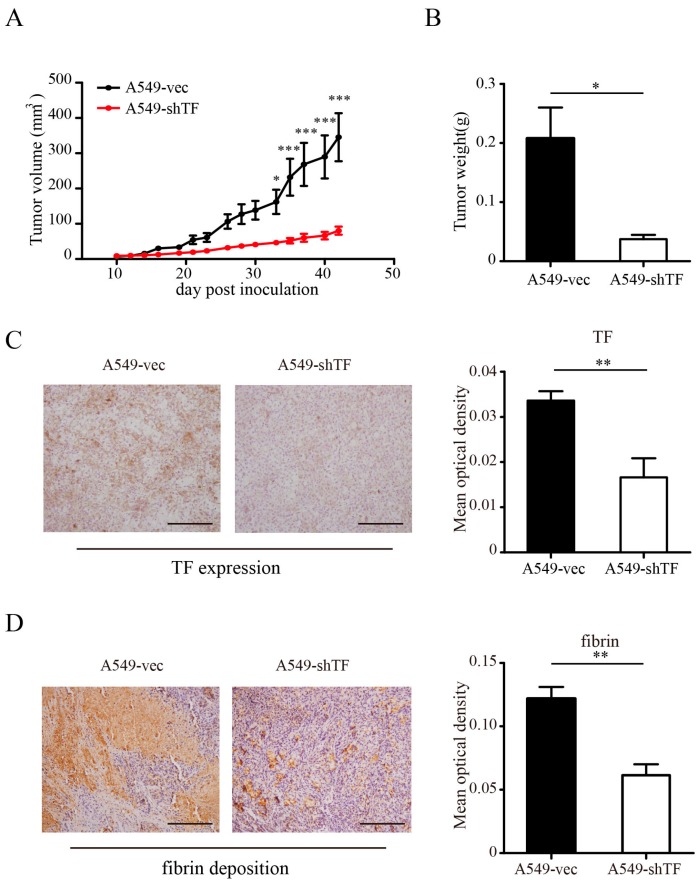
Effect of TF knockdown on tumor progression in vivo and local coagulation activation. A549-vec cells and A549-shTF cells were implanted into nude mice (*n* = 5) via subcutaneous injection. Tumor volume (**A**) and tumor weight (**B**) are expressed as mean ± SEM; (**C**) tumor sections in xenograft model were subjected to immunostaining analysis for TF expression, (**left** panel, bar = 100 μm) and (**D**) fibrin deposition, (**left** panel, bar = 100 μm) and (**C**) statistics of the mean optical density of TF, (**right** panel) and (**D**) fibrin staining, (**right** panel) were shown (*n* = 3). Data are expressed as mean ± SEM, * *p* < 0.05, ** *p* < 0.01, *** *p* < 0.001.

The coagulation activation triggered by TF requires the participation of molecules such as FVII and FX, among others, which are missing during in vitro cell culture, but are present in xenotransplant models. Thus, functional studies of TF in cultured cells are limited. To further assess the role of TF in tumor development in vivo, we subcutaneously inoculated 1.0 × 10^6^ A549-vec cells or A549-shTF cells into the right flank of nude mice. Tumor growth was monitored every other day. In contrast to our in vitro study, the tumor volume as well as tumor weight in the group of mice bearing tumors from A549-shTF cells were much smaller compared with those in the group of mice with A549-vec derived tumors (Figure 3A,B). TF expression in vivo was validated by immunostaining of TF in tumor sections (Figure 3C). We also examined the coagulation state of tumors by immunohistochemical analysis using a rabbit anti-mouse fibrin antibody. The staining showed massive fibrin deposition in the tumors, and in the tumors derived from A549-vec cells, the fibrin deposition was higher compared to that of tumors derived from A549-shTF cells (Figure 3D), suggesting more intense coagulation activation in the tumor derived from A549-vec cells.

### 2.4. Coagulation Induced by TF Activated Complement and Recruited MDSC

**Figure 4:**
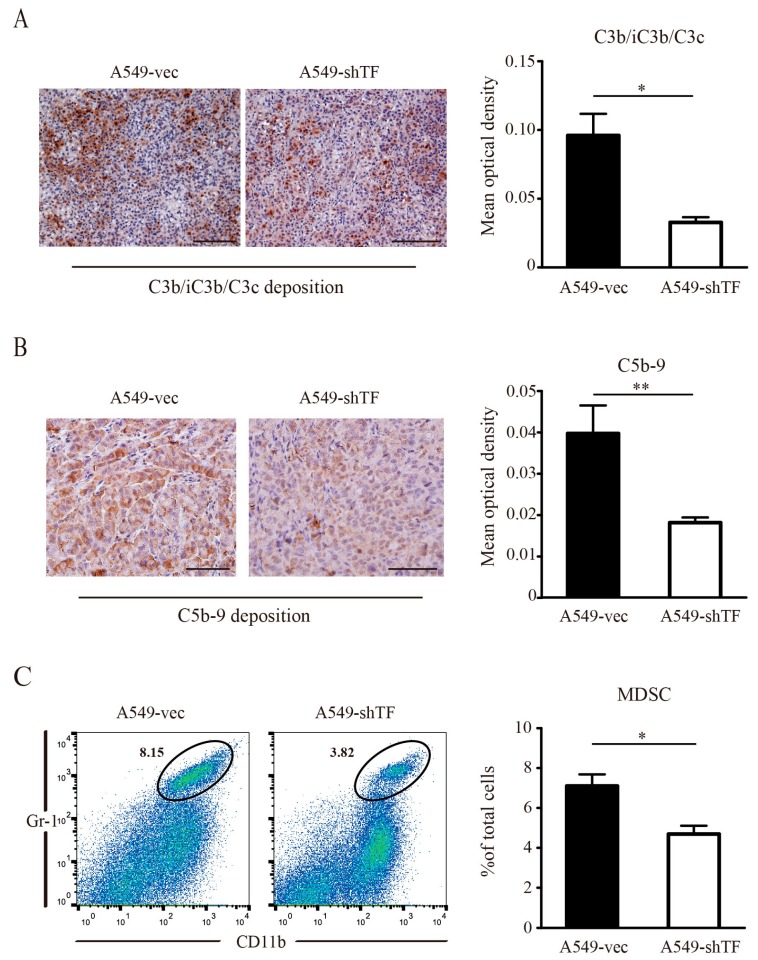
Effect of TF knockdown on complement C3b/iC3b/C3c and C5b-9 deposition and myeloid-derived suppressor cell (MDSC) recruitment in tumor microenvironment. (**A**) Immunostaining analysis of complement C3b/iC3b/C3c deposition, (**left** panel, bar = 100 μm) and (**B**) C5b-9, (**left** panel, bar = 50 μm) in paraffin section from tumor derived from A549-vec cells and A549-shTF cells (*n* = 3); (**A**) statistics of the mean optical density of C3b/iC3b/C3c staining, (**right** panel) and (**B**) C5b-9, (**right** panel) were shown, * *p* < 0.05, ** *p* < 0.01; (**C**) flow cytometry analysis of MDSC (gated as CD11b^+^ Gr-1^+^ in black circle) infiltration (**left** panel) in tumor derived from A549-vec cells and A549-shTF cells (*n* = 3); (**C**) statistics of the percentage of MDSCs in tumor (**right** panel), data are expressed as mean ± SEM, * *p* < 0.05, ** *p* < 0.01.

Coagulation has been thought to promote tumor progression, as it contributes to inflammatory events. Additionally, it directly cleaved complement factors in several disease models. To determine whether TF-induced coagulation contributed to complement activation within the tumor microenvironment, we measured complement activation by immunohistochemical analysis. Staining of C3b/iC3b/C3c revealed decreased C3b/iC3b/C3c deposition in tumor tissues derived from A549-shTF cells compared to tumor tissues derived from A549-vec cells (Figure 4A), suggesting that TF-induced coagulation facilitated complement activation within the tumor microenvironment. As thrombin can also cleave C5 in the absence of C3, we evaluated C5 cleavage by immunohistochemical analysis of C5b-9 deposition in tumor tissues, which indirectly reflects the cleavage of C5. The statistical results of C5b-9 staining revealed that the C5b-9 deposition in tumors from A549-shTF cell-bearing mice was lower compared to tumors from A549-vec cells (Figure 4B), which was consistent with the observed C3b/iC3b/C3c deposition. Previous studies have reported that complement facilitates tumor development. This protumor effect was mainly attributed to the release of anaphylatoxin C5a and subsequent myeloid-derived suppressive cells (MDSC) recruitment [16]. To validate this theory in our model, we evaluated the percentage of MDSC within tumor tissues. Compared with tumors from A549-vec cells, tumors grown from A549-shTF cells exhibited a decreased percentage of MDSC (Figure 4C), which could be explained by a decreased presence of anaphylatoxins due to insufficient complement activation.

### 2.5. C5a Receptor Antagonism Blunted the Effect of TF by Inhibiting MDSC Recruitment

**Figure 5:**
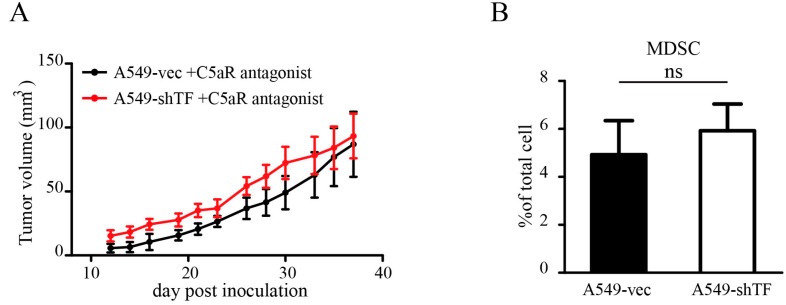
Effect of C5aR antagonism on tumor growth and MDSC recruitment. Tumor growth of A549-vec cells and A549-shTF cells injected subcutaneously in nude mice treated with a C5aR antagonist (*n* = 5). Tumor volume (**A**) is expressed as mean ± SEM. Percentage of MDSC (**B**) in tumor is analyzed by flow cytometry, ns, no significance.

Based on our findings, we tried to blunt the protumor effect of coagulation by C5aR antagonist administration to inhibit MDSC recruitment. Upon C5aR antagonist administration, the tumor growth in A549-vec cell-bearing mice was significantly impaired, and the tumor volume was equal to that of tumors derived from A549-shTF cells (Figure 5A). Additional evaluation of MDSC infiltration (Figure 5B) validated the observation that C5aR antagonist administration abolished the difference in MDSC infiltration between the two groups.

## 3. Discussion

Since Trousseau’s syndrome was first defined in 19th century, the association between aberrant coagulation and cancer has been discussed. The initiation of the coagulation cascade by TF-expressing cancer cells was considered to be the predominant cause of the cancer-induced hypercoagulant state in cancer patients. The overexpression of TF and its isoform, asTF, was found in a spectrum of different tumor types and was correlated with poor prognosis in clinical studies [5,8,24,25,26,27]. This dramatic upregulation of TF isoforms may be attributed to the existence of common stimuli from the tumor microenvironment, such as inflammation and hypoxia [5,28]. In addition, TF and asTF were indicated to play a role in tumor progression in several studies, indicating their role in promoting cell proliferation, tumor angiogenesis [5,27,29], and clot formation in the circulation [30,31], which was directly linked to cancer-associated VTE. Recently, researchers have begun to focus on the effect of TF in the tumor microenvironment. A study by Liu et al. suggested that the enhanced permeability of the tumor vasculature allowed soluble coagulant factors (e.g., FVII and FX) to be readily extravasated from the tumor vasculature into the tumor microenvironment and interact with TF, leading to local coagulation activation [11]. This study raised the concern of the procoagulant activity of tumor cells within the tumor microenvironment. In this study, we validated the dominant role of TF in the procoagulant activity of A549 cells in vitro before our in vivo experiments, which indicated that TF knockdown led to less coagulation of A549 cells and laid the foundation for our further experiments interfering with microenvironmental coagulation. This result is in agreement with a previous study that examined the procoagulant activity of tumor cells using an anti-TF antibody [21]. We also studied the biological characteristics of A549 cells after TF knockdown. The results showed that A549 cells both retained their proliferative potential and maintained their apoptosis rate in vitro despite TF knockdown. In contrast to our findings, several studies have reported that TF promotes tumor cell proliferation through protease-activated receptors (PARs) signaling and integrin signaling [5,32,33]. We believe this contradiction is partially explained by the lack of other coagulation cascade components in the culture medium, as PAR signaling activation requires the active forms of FX and thrombin [34], which are absent in cell culture. In support of this deduction, works from other researchers demonstrated that TF expression in tumor cells is dispensable for in vitro proliferation but specifically impacts the biology of tumor growth in vivo [23,35,36,37]. However, studies from Eisenreich et al. reported a novel signaling pathway that asTF could induce cell proliferation and angiogenesis via integrin signaling [5,8,27,29], thus it would be an attractive issue for researchers to reveal the precise role of these two signaling induced by TF and asTF in tumor biology.

In the traditional view, coagulation and complement are two conserved cascades that function separately. They share one common characteristic as being the innate serine protease system. Serine proteases are characterized by a serine residue at the active center of these proteases [38], which cleave arginyl-X peptide bonds in their substrates. Normally, C3 and C5 convertase individually cleave complement C3 and C5 at a single arginine-serine peptide in the α-chain to release C3a and C5a. Similarly, the interaction between TF and FVII results in a rapid and autocatalytic cleavage of a single peptide bond between arginine 152 and isoleucine 153 in the γ-carboxyglutamic acid (Gla) domain of FVII [39] to form TF-FVIIa, which transforms FX to FXa and subsequently induces thrombin generation and fibrin deposition. Recent studies have revealed more and more links between these two protease systems. In 1981, Kaplan et al. reported that human coagulation factor XIIa can activate the complement factor C1r, which can subsequently initiate the classical complement pathway [40]. Later, work from Peter et al. in an acute lung injury model proved that thrombin can cleave complement C5 in the absence of C3 [18], which was the first in vivo experiment supporting the concept of coagulation-induced complement activation. However, this particular form of complement activation in the tumor microenvironment had never been reported. In the current study, our data reveal that TF knockdown in A549 cells results in significantly decreased fibrin deposition in a xenotransplanted model, which reflected the coagulant state within the tumor microenvironment. The decreased fibrin deposition was accompanied with decreased C3b/iC3b/C3c and C5b-9 deposition, which represent the complement activation state. Considering the procoagulant activity of TF in A549 cells, it is not difficult to conclude that the coagulation triggered by TF in tumors contributes to complement activation, which is in line with previous studies on complement-coagulation interaction [18,19].

In our study, we found that TF-induced tumor progression could be blunted by C5aR antagonism, indicating that the influence of TF is complement dependent. The complement system is a part of the innate immune system that helps to clear abnormal host cells and pathogens via opsonization and cytolysis [13]. In particular, the cytolysis ability is facilitated by the assembly of C5b-9 on target cells, which is believed to have potential antitumor activities [41]. In 2008, John et al. first reported that the complement fragment C5a facilitates tumor growth by recruiting MDSC to the tumor site, confirming the dual roles of complement in tumors [16]. Consistent with this, our data suggest that increased MDSC infiltration is detected in tumors with more complement C3b/iC3b/C3c and C5b-9 deposition. Regardless to the cytolytic activity of C5b-9, we found that tumors derived from A549-vec cells grow larger despite more C5b-9 deposition. This may be due to the existence of a threshold amount of C5b-9 required for the induction of complement-dependent cytotoxicity. The cleavage of C5 simultaneously releases anaphylatoxin C5a, which recruits MDSC. Recent research has also emphasized that C5a is the pivotal element of the protumor effect of complement [42]. C5a antagonism was previously performed by Ruben et al. in 2012. In this experiment, the administration of a C5aR antagonist in immunocompetent mice bearing 3LL tumors reduced the MDSC population, which facilitated tumor development via promoting angiogenesis, but had no effect on T cells or Treg cells [17]. These findings indicate that the major target of the C5aR antagonist in tumor-bearing mice was MDSC, which could be a reasonable explanation for our observation that C5aR antagonism also worked in immunocompromised mice lacking mature T cells and functional Treg cells.

In summary, TF expression in malignant cells is widely recognized, yet its function in cancer development remains unclear. Our findings reveal that TF expressed by cancer cells facilitates complement activation in the tumor microenvironment via triggering the coagulation cascade, which is an essential contributor to tumor progression by recruiting MDSC. Herein, we showed that abrogation of the TF protumor effect can be achieved by the administration of a C5aR antagonist. Our findings provide novel evidence of coagulation-complement interaction within the tumor environment which may allow for the development of new therapeutic methods in tumor treatment.

## 4. Materials and Methods

### 4.1. Cell Lines and Culture

The human lung adenocarcinoma cell line A549, breast cancer cell line T47D, ovarian cancer cell line A2780, and gastric adenocarcinoma cell line AGS were purchased from American Type Culture Collection. These cells were cultured in RPMI-1640 medium (Gibco, Invitrogen Corporation, Grand Island, NY, USA), which was supplemented with 10% fetal bovine serum (Gibco) and 1% penicillin streptomycin solution (C0222, Beyotime, Shanghai, China). All cell lines were cultured at 37 °C in a 5% CO_2_ atmosphere.

### 4.2. Transfection

Green fluorescent protein (GFP)-expressing lentivirus and TF-shRNA lentivirus were purchased commercially (Genechem, Shanghai, China). Briefly, a stem-loop structure oligonucleotide containing the TF target sequence and a GFP reporter were cloned under the control of the human U6 promoter in lentiviral vectors. The sequence of the TF-shRNA is 5′-GCGCUUCAGGCACUACAAA-3′. The transfection of A549 cells was performed according to the manufacturer’s protocol. Briefly, a total of 5 × 10^4^ A549 cells were seeded into a 24-well plate. After cell adherence, 1 × 10^6^ IU of lentivirus was added to the culture medium, at a final Multiplicity of infection (MOI) of 20. The lentivirus-containing medium was maintained for 24 h before being replaced by fresh culture medium. The transfected cells were cultured normally and enriched by fluorescence-activated cell sorting (FACS). The efficiency of transfection was evaluated by real-time Polymerase chain reaction (rt-PCR) and Western blot analysis.

### 4.3. RNA Isolation and Real-Time Polymerase Chain Reaction

Total RNA was extracted from cells using Trizol reagent (Invitrogen, Life Technologies, Inc., Darmstadt, Germany). Then, 1 μg of isolated RNA was reverse transcribed into Complementary DNA (cDNA). For TF mRNA detection, the probe and corresponding primers were designed using the Universal ProbeLibrary online system (Roche Life Science, Basel, Switzerland) according to NCBI sequence NM_001993.3, and the expression level of β-actin (NM_001101.3) was used as an internal control. The thermocycler (Applied Biosystems, Foster City, CA, USA) conditions were as follows: an initial denaturation step at 95 °C for 10 min, followed by 45 cycles of 95 °C for 10 s and 60 °C for 30 s accompanied by fluorescence detection, and a final holding stage at 40 °C for 30 s. Each individual amplification was performed in triplicate. For statistical analysis, the Cycle Threshold (*C*_t_) of each target gene was normalized to the CT of β-actin (2^−Δ*C*t^) and the relative expression was calculated as the fold change relative to the control group (2^−ΔΔ*C*t^). All experiments were performed in triplicate.

### 4.4. Western Blot Analysis

Western blot analysis was performed according to a previously described protocol [43]. In brief, cells were resuspended in Radio Immunoprecipitation Assay (RIPA) lysis buffer (P0013B, Beyotime) containing 1 nM phenylmethane sulfonyl fluoride (PMSF) (ST506, Beyotime) and centrifuged at 12,000× *g* for 5 min at 4 °C. The supernatant was collected, mixed with sodium dodecyl sulfate-Polyacrylamide gel electrophoresis (SDS-PAGE) sample loading buffer (P0015, Beyotime), and boiled before electrophoresis. After protein transfer, polyvinylidene difluoride (PVDF) membranes (IPVH00010, Merck Millipore, Darmstadt, Germany) were blocked with Phosphate-buffered saline (PBS) (Zhongshan Goldenbridge Biotechnology Company, Beijing, China) containing 5% bovine serum albumin (BSA) and incubated with an anti-human TF (sc-80952, Santa Cruz Biotechnology Inc., Dallas, TX, USA) or anti-human β-actin (ab8226, Abcam, Cambridge, MA, USA) antibody overnight. The horseradish peroxidase (HRP)-conjugated secondary antibody (zb-5305, Zhongshan Biotechnology Co., Beijing, China) was diluted 1:5000 in Tris-buffered saline (pH = 7.4) plus 0.25% Triton-X100 (TBST). The HRP signal was visualized with an enhanced chemiluminescence system (BeyoECL Plus, Beyotime). The experiments were performed in triplicate.

### 4.5. Measurement of Prothrombin Time

A549 cells were harvested from the culture medium, and the concentration of the single-cell suspension was adjusted to 5 × 10^5^/mL, 1 × 10^6^/mL, 2 × 10^6^/mL, and 5 × 10^6^/mL. Whole blood from healthy volunteers was mixed with sodium citrate in a proportion of 9:1. The blood mixture was centrifuged at 3000 rpm for 10 min at room temperature to isolate plasma. The plasma was collected and incubated with the cell suspension in different concentrations at 37 °C for 30 min. The mixture was then sent for prothrombin time detection using a BSC XP system (Siemens Healthcare GmbH, Erlangen, Germany) with an additional 12.5 mM CaCl_2_ in Hanks Balanced Salt Solutions reagent. The prothrombin time of A549-vec and A549-shTF cells was measured using the same procedure. The study received the approval of the Institutional Review Board of Xinqiao Hospital and informed consent was obtained from all volunteers. These experiments were performed in triplicate.

### 4.6. In Vivo Xenograft Experiments

Nude mice (six to eight weeks old, female) were purchased from the Chinese Academy of Medical Sciences (Beijing, China) and fed in specific pathogen-free (SPF) conditions. For xenograft generation, 1 × 10^6^ A549-vec or A549-shTF cells suspended in 100 μL PBS (Zhongshan Goldenbridge Biotechnology Company) were subcutaneously injected into the right flank of nude mice (*n* = 5). For the C5aR antagonist experiment, A549-vec or A549-shTF cells were injected as described above. Mice (*n* = 5) were administered a C5aR antagonist (GL Biochem, Shanghai, China) by daily intraperitoneal injection at a concentration of 3 mg/kg. Tumor growth was monitored by measuring with Vernier calipers, and the tumor volume was calculated using the formula (length × width^2^ × 0.5). The tumor tissues were then subjected to immunohistochemical and flow cytometric analyses. The animal study was approved by Laboratory Animal Welfare and Ethics Committee of the Third Military Medical University (SYXK-PLA-20120031, 15 January 2014). All in vivo experiments were performed in triplicate.

### 4.7. Immunohistochemical Analysis

Tumors were excised, fixed in 4% formalin (Zhongshan Goldenbridge Biotechnology Company) for approximately 18–24 h, embedded in paraffin, and sliced in 6-μm sections. Antigen retrieval was performed by heating the sample in a microwave for 20 min with 10 mM sodium citrate (pH = 6.0). The endogenous peroxidase was quenched by adding the hydrogen peroxide (3% H_2_O_2_, (Zhongshan Goldenbridge Biotechnology Company)) at room temperature for 15 min and the sections were then blocked with 5% bovine serum albumin (BSA) for 1 h. The sections were incubated with the indicated antibody overnight at 4 °C (anti-human TF monoclonal antibody (sc-80952, Santa Cruz Biotechnology Inc., Dallas, TX, USA), anti-mouse C5b-9 monoclonal antibody (204903, Calbiochem, San Diego, CA, USA), anti-mouse C3b/iC3b/C3c monoclonal antibody (HM1065, Hycult Biotech, Uden, The Netherlands), and anti-mouse fibrin polyclonal antibody (F0111, Agilent Technologies, Santa Clara, CA, USA)). For negative controls, sections were stained with PBS (Zhongshan Goldenbridge Biotechnology Company) instead of the primary antibodies. The sections were then washed with PBS (Zhongshan Goldenbridge Biotechnology Company) and incubated with HRP-conjugated goat anti-mouse IgG (Zhongshan Goldenbridge Biotechnology Company) for 30 min at 37 °C. 3,3-diaminobenzidine (DAB) (Beyotime) and hematoxylin (Beyotime) were used for counterstaining, and the sections were sealed with coverslips, fixed with resin (Zhongshan Goldenbridge Biotechnology Company), and submitted for examination. The tumor sections were randomly photographed in a high-power field (200× or 400×) in a blinded fashion without knowledge of tissue source. The semiquantitative analysis of positive staining was performed using ImagePro Plus (IPP) software (Media Cybernetics, Warrendale, PA, USA). Analysis of the positive staining from each image was performed after calibration of the light intensity to ensure background subtraction. The value of integrated optical density (IOD) of positive staining and the total of area of interest (AOI) of each image were measured individually using IPP software (Media Cybernetics, Warrendale, PA, USA). The mean optical density (MOD) of each image was calculated according to the formula: MOD = IOD/AOI. Quantitative image analysis data obtained from five random fields of each section were averaged for each sample and subjected for statistical analysis. All experiments were performed in triplicate.

### 4.8. Flow Cytometric Analysis

For flow cytometry analysis, tumor masses and spleens were prepared by mechanical dissociation in a 40-μm filter and suspended in FACS buffer (PBS (Zhongshan Goldenbridge Biotechnology Company) containing 0.05% ethylenediaminetetraacetic acid (EDTA) (Beyotime Inst Biotech, Shanghai, China)). For each individual test, the amount of cells was adjusted to approximately 10^6^, and 1 mL ACK Lysing Buffer (Beyotime Inst Biotech) was added to each test for red blood cell clearance. For MDSC detection, FITC-conjugated CD45 (Biolegend, San Diego, CA, USA), APC-conjugated CD11b (Biolegend), and Percp-cy5.5-conjugated Lymphocyte antigen 6G/Lymphocyte antigen 6C (Gr-1) (Biolegend) were used as MDSC labels, and the procedures as well as dilutions were performed according to the manufacturer’s instructions. A matched isotype antibody (Rat IgG2b, Biolegend) was used as a negative control. For GFP-positive cell sorting, cell suspensions were harvested from culture dishes, and the sorting was conducted on a FACSAria II cell sorter (BD Biosciences, San Jose, CA, USA). For Ki-67 detection, A549, A549-vec, and A549-shTF cells were collected from six-well plates and dissociated into single cells. The Ki-67 staining was performed using the Foxp3/Transcription Factor Staining Buffer Set and an anti-human Ki-67 antibody (556027, BD Biosciences) based on the suggested protocol. For apoptosis analysis, A549, A549-vec, and A549-shTF cells were harvested and resuspended to single-cell suspensions. The Annexin V and PI staining were performed with an Annexin V Apoptosis Detection Kit FITC (88-8005-72, eBioscience, San Diego, CA, USA) according to the manufacturer’s protocol. All samples were washed and resuspended in FACS buffer, and flow cytometry was performed on a FACSCanto™ II instrument (BD Biosciences). FlowJo software (version 10.0; Tree Star Inc., San Diego, CA, USA) was used for all flow cytometry analyses. All experiments were performed in triplicate.

### 4.9. Cell Proliferation Assay

To analyze the proliferative ability of A549-shTF and A549-vec cells, a Cell Counting Kit-8 (CCK-8) (Beyotime Inst Biotech) was used according to the manufacturer’s instructions. Briefly, 2 × 10^3^ cells were seeded in a 96-well plate with another four replicants adding 100 mL RPMI-1640 containing 10% FBS and cultured for 24 h at 37 °C with 5% CO_2_. CCK-8 (10 μL) was added to each well, and the optical density (OD_450_) values were measured 2 h later by an enzyme-labeled instrument. This experiment was repeated in triplicate.

### 4.10. Statistics

All data are presented as mean ± SEM. The statistical significance of the data was determined by the two-tailed Student’s *t*-test and the statistical significance of tumor growth curve was determined by two way ANOVA.
